# Discrete Li-occupation versus pseudo-continuous Na-occupation and their relationship with structural change behaviors in Fe_2_(MoO_4_)_3_

**DOI:** 10.1038/srep08810

**Published:** 2015-03-06

**Authors:** Ji-Li Yue, Yong-Ning Zhou, Si-Qi Shi, Zulipiya Shadike, Xuan-Qi Huang, Jun Luo, Zhen-Zhong Yang, Hong Li, Lin Gu, Xiao-Qing Yang, Zheng-Wen Fu

**Affiliations:** 1 Shanghai Key Laboratory of Molecular Catalysts and Innovative Materials, Department of Chemistry & Laser Chemistry Institute, Fudan University, Shanghai 200433, P.R. China; 2 Departmentof Chemistry, Brookhaven National Laboratory, Upton, New York 11973, USA; 3 School of Materials Science and Engineering, Shanghai University, Shanghai 200444, P.R. China; 4 Beijing National Laboratory for Condensed Matter Physics, Institute of Physics, Chinese Academy of Sciences, PO Box 603, Beijing 100190, P.R. China

## Abstract

The key factors governing the single-phase or multi-phase structural change behaviors during the intercalation/deintercalation of guest ions have not been well studied and understood yet. Through systematic studies of orthorhombic Fe_2_(MoO_4_)_3_ electrode, two distinct guest ion occupation paths, namely discrete one for Li and pseudo-continuous one for Na, as well as their relationship with single-phase and two-phase modes for Na^+^ and Li^+^, respectively during the intercalation/deintercalation process have been demonstrated. For the first time, the direct atomic-scale observation of biphasic domains (discrete occupation) in partially lithiated Fe_2_(MoO_4_)_3_ and the one by one Na occupation (pseudo-continuous occupation) at 8d sites in partially sodiated Fe_2_(MoO_4_)_3_ are obtained during the discharge processes of Li/Fe_2_(MoO_4_)_3_ and Na/Fe_2_(MoO_4_)_3_ cells respectively. Our combined experimental and theoretical studies bring the new insights for the research and development of intercalation compounds as electrode materials for secondary batteries.

Intercalation compounds as energy storage materials have been extensively studied for secondary batteries^1^,^2^,^3^,^4^. All of these intercalation materials for the secondary batteries allow the guest ions to move in and out without significant damage of their host frameworks. The composition variations in intercalation compounds during the intercalation/deintercalation of guest ions are often accompanied by structural changes^5^,^6^. Most of intercalation compounds (Supplementary Table 1) fall into the single-phase solid solution mode^7^ or the two-phase transformation mode^8^ or three-phase separation mode^9^ as a result of the composition variations in the certain concentration range of guest ions. For example, the layered Li*_x_*CoO_2_ (0.5 < *x* ≤ 0.75) deintercalates/intercalates Li^+^ via a single-phase process^7^,^10^, while the layered Na*_x_*CoO_2_ (0.5 ≤ *x* ≤ 1) shows various single-phase or two-phase domains depending on Na^+^ concentration^11^. The olivine-type LiFePO_4_ exhibits a two-phase transformation reaction (LiFePO_4_/FePO_4_) by undergoing a phase interface propagation based on steady-state results^12^, and some non-equilibrium single-phase solid solution processes as predicted by *ab* initio calculations^13^ and confirmed by *in situ* diffraction experiments^14^,^15^. The various phase transformation mechanisms of Li^+^ ions in LiFePO_4_/FePO_4_ are revealed to be dependent on the rate^16^. Understanding these structural change mechanisms during the intercalation/deintercalation process is very important for the development of high energy density and long cycle-life batteries. Here, through the systematic studies of an intercalation compound of Fe_2_(MoO_4_)_3_, we report the single-phase structural change behavior for Na^+^ intercalation/deintercalation, but two-phase reaction mode for Li^+^ intercalation/deintercalation. The framework structure remains unchanged in the entire concentration range of Na^+^ or Li^+^. More interestingly, such single-phase and two-phase reactions are closely related to the guest ion occupation paths during intercalation/deintercalation, as clearly demonstrated by the aberration-corrected scanning transmission electron microscopy (STEM) results. These results provide new insights into the origin of structural changes in the guest-host material systems.

Fe_2_(MoO_4_)_3_ is one of the most promising cathode materials for rechargeable lithium/sodium battery as an environment friendly energy storage material from the viewpoints of the inexpensive and non-toxic of iron. From X-ray diffraction studies, it is known that Fe_2_(MoO_4_)_3_ has two types of crystal structures: low temperature monoclinic structure and high temperature orthorhombic structure. Although there have been several reports on the monoclinic Fe_2_(MoO_4_)_3_ as the cathode materials for lithium (or sodium) battery, the Li^+^ (or Na^+^) intercalation/deintercalation mechanisms remain unclear or contradict with each other. For example, some of literatures^17^,^18^,^19^,^20^ indicated a two-phase reaction during the intercalation/deintercalation of both Na^+^ and Li^+^ into the monoclinic Fe_2_(MoO_4_)_3_, whereas single-phase solid solution reaction of Na*_x_*Fe_2_(MoO_4_)_3_ (0 < *x* < 2) was also observed^21^. This may be due to the structural complex or thermodynamic unfavorableness of monoclinic Fe_2_(MoO_4_)_3_. In this work, orthorhombic Fe_2_(MoO_4_)_3_ was studied as the cathode material for lithium and sodium batteries. Its electrochemical properties and structural change behaviors during charge and discharge processes are investigated by synchrotron based X-ray diffraction (XRD), X-ray absorption spectroscopy (XAS), aberration-corrected scanning transmission electron microscopy (STEM) and first-principles thermodynamic calculations. The discrete Li occupation path and pseudo-continuous Na occupation path in Fe_2_(MO_4_)_3_ during intercalation/deintercalation process and their relationship with the two-phase and single-phase reactions are proposed.

## Results

### Electrochemical characterization

**Figure 1:**
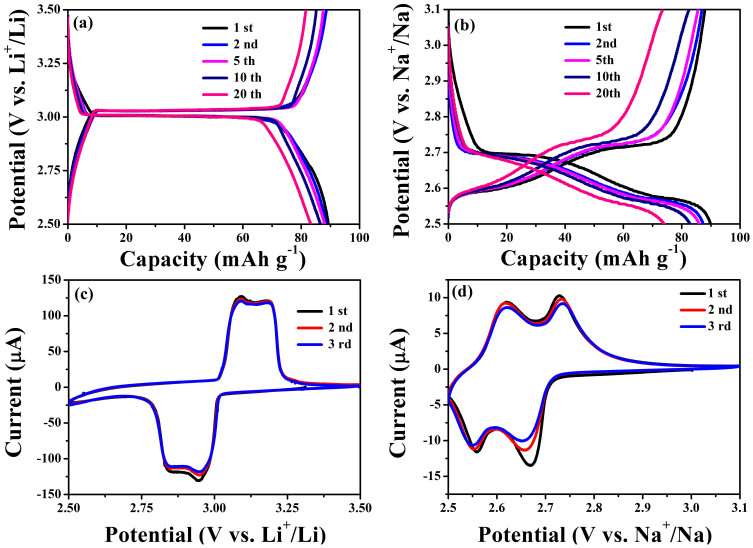
Electrochemical performance of Fe_2_(MoO_4_)_3_ in lithium and sodium cells. Galvanostatic discharge/charge curves of Fe_2_(MoO_4_)_3_ electrode under a current density of C/20 in (a) Li and (b) Na cells. Cyclic voltammogram curves of Fe_2_(MoO_4_)_3_ electrode in the initial three cycles at the scan rate of 0.01 mVs^−1^ in (c) lithium and (d) sodium cells.

The intercalation/deintercalation behaviors of alkali (*A* = Li or Na) metal ions in the Fe_2_(MoO_4_)_3_ were examined in Li and Na cells in Fig. 1. As shown in Fig. 1a and b, the initial discharge capacity of 89.5 mAh g^−1^ can be obtained for both Li and Na cells at the current rate of C/20. This value corresponds to the intercalation number of 2.0 Li or Na per Fe_2_(MoO_4_)_3_ unit. The discharge/charge curves in the lithium cell (Fig. 1a) show a flat plateau at about 3.0 V vs. Li^+^/Li during the discharging process and a flat plateau at about 3.02 V vs. Li^+^/Li during the charging process in a large range. By contrast, the discharge/charge curves of Na/Fe_2_(MoO_4_)_3_ cell show a slope type in the voltage range of 2.5 to 2.7 V vs. Na^+^/Na in Fig. 1b. The capacity fades of Li/Fe_2_(MoO_4_)_3_ and Na/Fe_2_(MoO_4_)_3_ cells during the first 20 cycles are about 0.3% and 0.9% per cycle, respectively, indicating a better capacity retention of Li/Fe_2_(MoO_4_)_3_ cell than that of Na/Fe_2_(MoO_4_)_3_ cell. The discharge and charge curves of Li/Fe_2_(MoO_4_)_3_ cell at a current density of C/5 shown in Supplementary Fig. 1 indicates a good cyclic performance up to 400 cycles with a capacity fading less than 0.02% per cycle. The shapes of one pairs of cathodic peak and anodic peak in the cyclic voltammogram (CV) curves of Li/Fe_2_(MoO_4_)_3_ cell exhibit the feature of mirror-symmetry as shown in Fig. 1c. Such an appearance of the peak is related to the typical diffusion and reaction kinetics at around half-discharging and half-charging processes. For the CV curves of Na/Fe_2_(MoO_4_)_3_ cell, two couples of reduction/oxidation peaks at around 2.65/2.73 V and 2.54/2.62 V (Fig. 1d) are in good agreement with the discharge/charge curves.

### *Ex situ* XRD patterns

**Figure 2:**
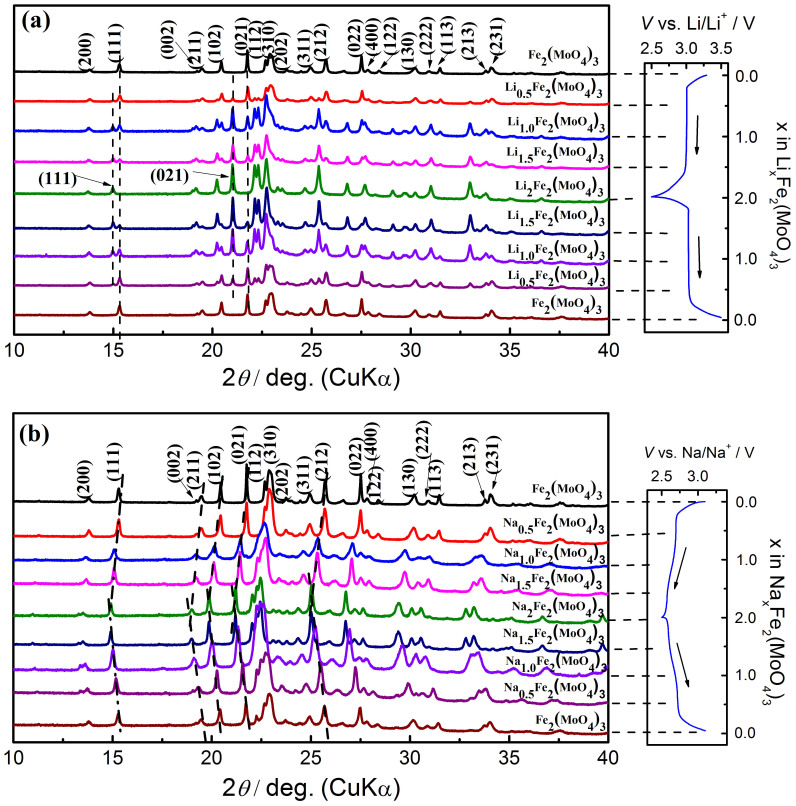
*Ex situ* XRD patterns of Fe_2_(MoO_4_)_3_ at different discharge and charge states. (a) Li and (b) Na cells during the initial cycle (the 2 theta is converted to corresponding angle for λ = 1.54 Å (Cu-Kα) from the real wavelength λ = 0.7747 Å used for synchrotron XRD experiments).

To investigate the structural evolutions of Fe_2_(MoO_4_)_3_ during Li^+^ and Na^+^ intercalation/deintercalation, a series of synchrotron based XRD patterns were collected at different charge/discharge states. As shown in Fig. 2a for Li*_x_*Fe_2_(MoO_4_)_3_ (*x* = 0.0, 0.5, 1.0, 1.5 and 2.0) during the first discharge-charge cycle in a Li/Fe_2_(MoO_4_)_3_ cell. All diffraction peaks of the pristine and fully lithiated Fe_2_(MoO_4_)_3_ (after discharging to 2.5 V) can be well indexed to Fe_2_(MoO_4_)_3_ (JCPDS card No. 852278) and Li_2_Fe_2_(MoO_4_)_3_ (JCPDS card No. 841001) with the same orthorhombic structure, respectively. During the discharge process, the intensity of the major peaks (111), (211), (012), (021), (310), (212) and (231) representing Fe_2_(MoO_4_)_3_ decreased gradually and finally disappeared when the 2.5 V discharge limit was reached. At the meantime, a new Li_2_Fe_2_(MoO_4_)_3_ phase was formed as observed through the appearing and growing intensity of a new set of (111), (211), (012), (021), (310), (212) and (231) peaks at lower *2θ* angles relative to those original ones. No peak shifts are observed for both Fe_2_(MoO_4_)_3_ and Li_2_Fe_2_(MoO_4_)_3_ phases in the entire discharge process. During the recharge process, the peak evolution is exactly in the opposite way to the discharge process. After the initial cycle, the Fe_2_(MoO_4_)_3_ phase can be fully recovered, indicating an excellent structural reversibility. The coexistence of both Fe_2_(MoO_4_)_3_ and Li_2_Fe_2_(MoO_4_)_3_ phases with changing ratio, demonstrates a typical two-phase reaction during the discharge and charge process in the lithium cell.

Fig. 2b shows the XRD patterns of Na*_x_*Fe_2_(MoO_4_)_3_ (*x* = 0.0, 0.5, 1.0, 1.5 and 2.0) during the first discharge-charge cycle in a Na/Fe_2_(MoO_4_)_3_ cell. Interestingly, a completely different structural change behavior other than that in the lithium cell was observed. No new set of peaks, but only peak shifts were observed throughout the entire discharge/charge process. During the discharge process, major peaks (111), (211), (102), (021), (310), (212), (022), (130) and (231) all gradually moved toward lower *2θ* angles with increasing *x* from 0.0 to 2.0 in Na*_x_*Fe_2_(MoO_4_)_3_. During the recharge process, all of these peaks reversibly moved back to their original positions with decreasing *x* from 2.0 to 0.0. The reversible peak shifts are attributed to the continuous lattice expansion and contraction during the discharge and charge respectively. This result demonstrates a typical single-phase (solid-solution) reaction during the discharge and charge process in the sodium cell.

### DFT simulations

**Figure 3:**
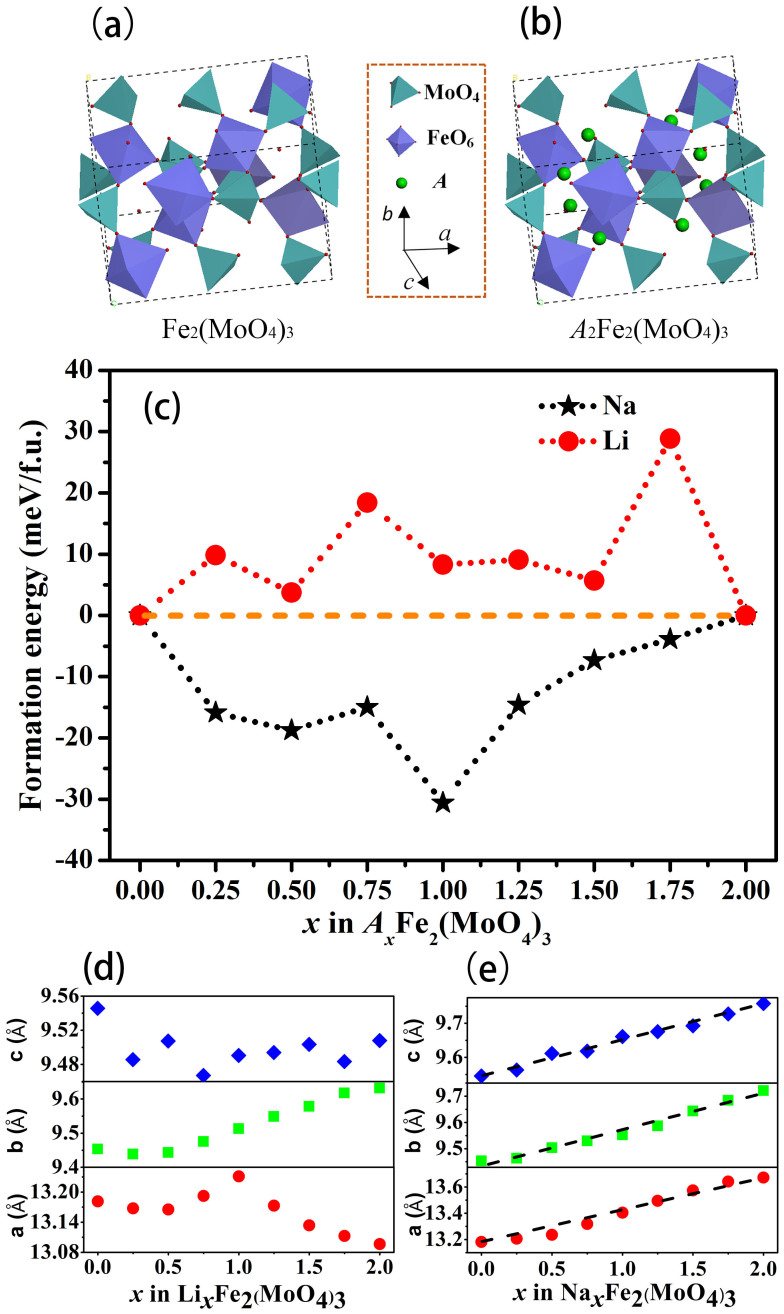
DFT simulations. Crystal structures of (a) Fe_2_(MoO_4_)_3_ and (b) *A*_2_Fe_2_(MoO_4_)_3_. (c) Formation energies of *A_x_*Fe_2_(MoO_4_)_3_ at different *x* values (*A* = Li or Na). Optimized lattice parameters of (d) Li*_x_*Fe_2_(MoO_4_)_3_ and (e) Na*_x_*Fe_2_(MoO_4_)_3_ at different *x* values.

It is quite interesting to note that the same Fe_2_(MoO_4_)_3_ orthorhombic structure shows such different structural change behaviors during Li^+^ and Na^+^ intercalation/deintercalation process. The crystal structure and thermodynamics of the orthorhombic Fe_2_(MoO_4_)_3_ during guest alkali ion insertion were further studied. As shown in Fig. 3a and b, the crystal structure of the orthorhombic Fe_2_(MoO_4_)_3_ with a space group of Pbcn is composed of MoO_4_ tetrahedra sharing all four corners with FeO_6_ octahedra and FeO_6_ octahedra sharing all six corners with MoO_4_ tetrahedra. Such an open three dimensional framework structure is suitable for guest (*A* = Li and Na) ions accommodation and diffusion. After the alkali ion intercalation, the crystal structure of Li_2_Fe_2_(MoO_4_)_3_ is isostructural to Na_2_Fe_2_(MoO_4_)_3_ based on *ab initio* calculations. *A*_2_Fe_2_(MoO_4_)_3_ (*A* = Li, Na) have the same orthorhombic structure as Fe_2_(MoO_4_)_3_. The eight *A* ions occupy the 8d tetrahedra interstitial sites in a unit cell. Their lattice parameters and unit cell volumes obtained from Le Bail fitting of the XRD patterns are listed in Supplementary Table 2. In order to understand the thermodynamic origin about the intercalation/deintercalation behaviors of *A* ions in the Fe_2_(MoO_4_)_3_, the formation energy (*E*_f_) of *A_x_*Fe_2_(MoO_4_)_3_ with respect to Fe_2_(MoO_4_)_3_ and *A*_2_Fe_2_(MoO_4_)_3_ via: 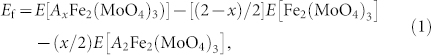
where three energy terms represent the total energies of per *A_x_*Fe_2_(MoO_4_)_3_, Fe_2_(MoO_4_)_3_ and *A*_2_Fe_2_(MoO_4_)_3_ formula unit (f.u.), respectively. Here a completely random *A*/vacancy distribution at 8d site in *A_x_*Fe_2_(MoO_4_)_3_ is given, the configurational entropy, *S*_con_ = *k*_B_[(*x*/2)ln(*x*/2) + (1 − *x*/2)ln(1 − *x*/2)], is included in the total energy of *A_x_*Fe_2_(MoO_4_)_3_. To obtain the most energetically favorable configuration, all possible *A* occupancies were considered. There are 248 possibilities in all, namely, 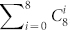, where 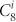 (*i* = 0, 1, 2, 3, 4, 5, 6, 7, 8) stands for possible arrangements of different numbers of *A* in a unit cell (*Z* = 4). Only the calculated lowest formation energies of *A_x_*Fe_2_(MoO_4_)_3_ at different *x*(*x* = *i*/Z) values are shown in Fig. 3c. It can be found that the single-phase solid solution of Li*_x_*Fe_2_(MoO_4_)_3_ is not thermodynamically favorable with the positive formation energies of 5~30 meV/f.u., which implies the phase separation of Fe_2_(MoO_4_)_3_ and Li_2_Fe_2_(MoO_4_)_3_. Conversely, Na*_x_*Fe_2_(MoO_4_)_3_ exhibits the negative formation energies (−12~−40 meV/f.u.), which is representative of the single-phase solid solution instead of the two-phase separation. Dependences of calculated lattice parameters (*a*, *b* and *c*) on *x* in *A_x_*Fe_2_(MoO_4_)_3_ are shown in Fig. 3d and e. Results indicate that the variation of the lattice parameters with *x* in Li*_x_*Fe_2_(MoO_4_)_3_ disobeys Vegard's law, thus invalidating the solid solution reaction for the lithium ion intercalation process. Nonetheless, the Vegard's law is true for the case of Na*_x_*Fe_2_(MoO_4_)_3_, indicating the single phase solid solution reaction for the sodium ion intercalation process. This suggests that the first-principles thermodynamic calculations draw the identical conclusion as the *ex* situ XRD patterns in Figure 2.

Considering an interface between Li_2_Fe_2_(MoO_4_)_3_ and Fe_2_(MoO_4_)_3_ feasibly forms with the lithium ion intercalation into Fe_2_(MoO_4_)_3_ but no interface exists with the sodium ion intercalation into Fe_2_(MoO_4_)_3_, an *A*_2_Fe_2_(MoO_4_)_3_/Fe_2_(MoO_4_)_3_ interface parallel to (010) plane (Supplementary Fig. 2) was constructed and the interfacial energy was calculated^22^. The interfacial energy, *γ*_interface_, is defined as *γ*_interface_ = (*E*_bulk_ − *E_A_*_,contained_ − *E_A_*_,free_)/2*S*, where *E*_bulk_ is the total energy of the given interface supercell, and *E_A_*_,contained_ and *E_A_*_,free_ are the total energies of the relaxed free *A*_2_Fe_2_(MoO_4_)_3_ and Fe_2_(MoO_4_)_3_ (010) surfaces. *γ*_interface(Li)_ and *γ*_interface(Na)_ were calculated to be −1.422 and −1.071 Jcm^−2^, respectively. It is very interesting to find that *γ*_interface(Li)_ is more negative than *γ*_interface(Na)_, indicating that Li_2_Fe_2_(MoO_4_)_3_/Fe_2_(MoO_4_)_3_ interface is more thermodynamically stable than Na_2_Fe_2_(MoO_4_)_3_/Fe_2_(MoO_4_)_3_ one. This result can be used for the explanation on the coexistence of two-phases and single-phase during the discharge processes of Li/Fe_2_(MoO_4_)_3_ and Na/Fe_2_(MoO_4_)_3_ cells, respectively.

### STEM imaging

**Figure 4:**
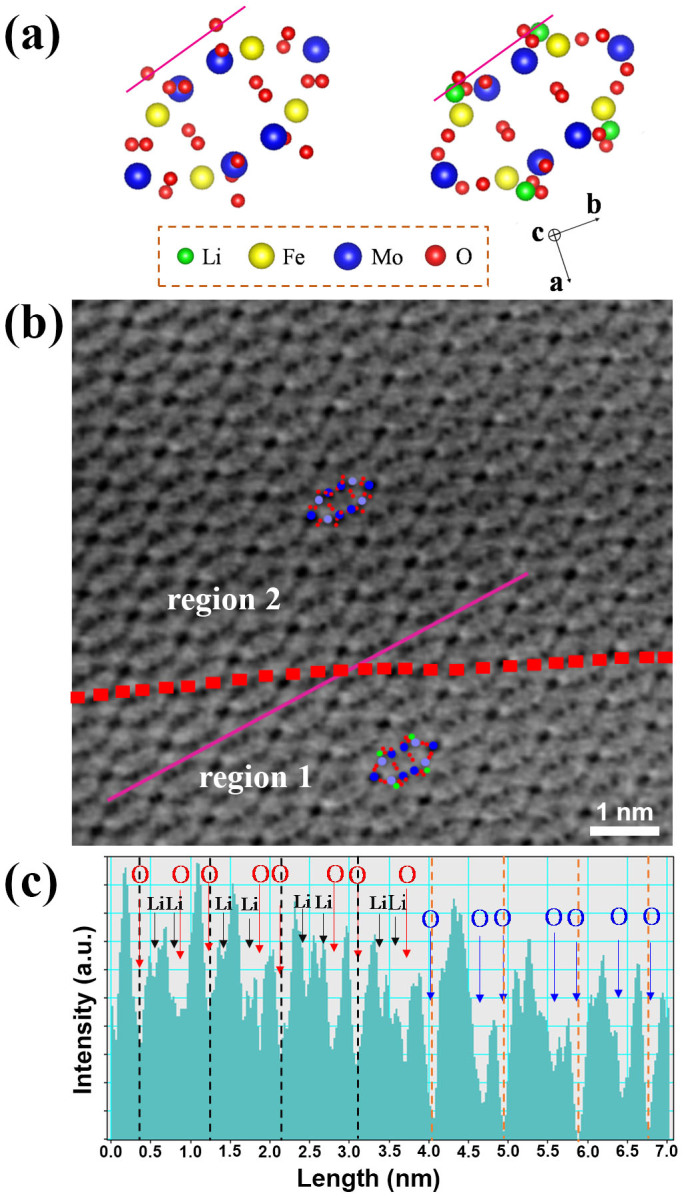
STEM images of half lithiated Fe_2_(MoO_4_)_3_. (a) Schematics of Fe_2_(MoO_4_)_3_ and Li_2_Fe_2_(MoO_4_)_3_ along the [001] projection. (b) ABF-STEM image of partially lithiated Fe_2_(MoO_4_)_3_ at the 1/2 discharge state. Atomic arrangements of Li_2_Fe_2_(MoO_4_)_3_ and Fe_2_(MoO_4_)_3_ are shown as the insets in the regions 1 and 2, respectively. (c) The corresponding ABF line profile acquired at the purples lines across the boundary (red dash lines) in (b). O sites and Li sites are marked by red and black arrows in the region 1 respectively, and the O sites are marked by blue arrows in the region 2.

To further confirm the two-phases and single-phase during the lithiation and sodiation processes of Fe_2_(MoO_4_)_3_, spherical aberration-corrected STEM were employed to obtain a direct observation at the atomic resolution. Considering the relative less structural stability of lithium-containing compound based on our own experience and reported works^23^. Here we decreased the probe current to about 20 pA and the pixel dwell time to 10 μs, to avoid the electron beam damage or phase transformation during STEM analysis. The schematic drawings for Fe_2_(MoO_4_)_3_ and Li_2_Fe_2_(MoO_4_)_3_ projected along the [001] direction have the ellipse-shaped unit constructions as shown in Fig. 4a. All annular-bright-field (ABF) images of partially lithiated Fe_2_(MoO_4_)_3_ at the 1/4, 1/2 and 3/4 discharge states were examined and the same two different regions could be observed in these ABF images. The typical ABF image of partially lithiated Fe_2_(MoO_4_)_3_ at the 1/2 discharge state is shown in Fig. 4b, in which one boundary is marked with a red dash line between regions 1 and 2. Unfortunately, lithium ions which are supposed to occupy both sides of the shoulder of ellipse cannot be obviously visualized because of the wide atomic number gap between Li and Mo. Therefore, line profiles (Fig. 4c) were acquired across two regions through the purple line in the ABF image (Fig. 4b) to confirm the lithium contrast with respect to oxygen. The corresponding purple lines in the Fe_2_(MoO_4_)_3_ and Li_2_Fe_2_(MoO_4_)_3_ unit structures are also shown in Fig. 4a. The Li and O positions are displayed as arrows in the line profile (Fig. 4c). The corresponding line profile shows two distinctly different periodic characteristics in these two regions. Region 1 shows the same featured line profiles as Li_2_Fe_2_(MoO_4_)_3_, in which O and Li can be well marked by red and black arrows, respectively (Fig. 4c) and four Li 8d sites occupy both sides of the shoulder of ellipse with symmetrical distribution close to four Mo 8d sites as shown in the unit structure of Li_2_Fe_2_(MoO_4_)_3_. In contrast, the line profiles in the region 2 are found to be the same as those of Fe_2_(MoO_4_)_3_ (Supplementary Fig. 3). After examining a group of line profiles (see Supplementary Fig. 4) in Fig. 4b, an interface of the two different phases can be marked with the red line in Fig. 4b. The coexistence of Li_2_Fe_2_(MoO_4_)_3_ and Fe_2_(MoO_4_)_3_ phases observed from the ABF image of the partially lithiated Fe_2_(MoO_4_)_3_ agrees very well with the XRD results in Fig. 2a.

**Figure 5:**
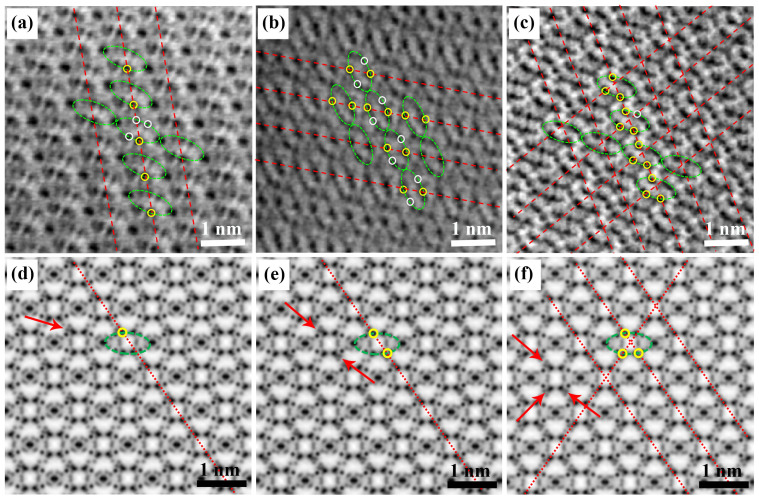
The STEM images of partially sodiated Fe_2_(MoO_4_)_3_. (a) at the 1/4, (b) at the 1/2 and (c) at the 3/4 discharged states viewed along [001] projection and their corresponding simulated ABF images (d), (e) and (f). Repeated unit structures are labeled by green ellipse. The Na occupied sites are marked by yellow circles and the unoccupied sites are marked by white circles in (a), (b) and (c).

ABF images of partially sodiated Fe_2_(MoO_4_)_3_ at the 1/4, 1/2 and 3/4 discharged states were also carefully examined, but only one uniform region is found in all of these images. As shown in Fig. 5 a, b and c, the repeat unit can be clearly visualized (shown in the green ellipses) in these ABF images, which has the identical cage structure with Fe_2_(MoO_4_)_3_. In the ABF images of sodiated Fe_2_(MoO_4_)_3_ at the 1/4, 1/2 and 3/4 discharged states, four spots representing Mo 8d sites on the shoulder of ellipse do not exhibit the same blackness as that in the pristine Fe_2_(MoO_4_)_3_ (Supplementary Fig. 3). Therefore, some intriguing contrasts of four black spots representing Mo 8d sites on the shoulder of ellipse between the pristine and partially sodiated Fe_2_(MoO_4_)_3_ in ABF images could provide the information of Na occupancy in Fe_2_(MoO_4_)_3_. Interestingly, one spot marked by yellow circle on the green ellipse is the best blackness in four spots representing Mo 8d sites on the shoulder of ellipse at the 1/4 discharge states, in which other three dark spots were marked by white circles (Fig. 5a). According to the atomic occupancies of simulated ABF image of Na_0.5_Fe_2_(MoO_4_)_3_ in Fig. 5d based on the energetically most favorable configuration (Supplementary Fig. 5), one Na 8d site (shown as the red arrow in Fig. 5d) resides in the vicinity of the one of four Mo 8d sites on the shoulder of ellipse in the construction of Na_0.5_Fe_2_(MoO_4_)_3_, resulting in that one of four spots representing Mo 8d sites at every repeated unit structure exhibits more blackness than any other three spots. The Na columns as red dash lines with the periodicity and homogeneity can be clearly observed in the ABF image (Fig. 5a). After the depth of discharge to 1/2, two spots representing Mo 8d sites (yellow circles) on both sides of the shoulder of ellipses with asymmetric distribution are found to be blacker than other two spots (white circles) as shown in Fig. 5b, which perfectly coincides with simulated ABF image of NaFe_2_(MoO_4_)_3_ structure based on first principle calculations (Supplementary Fig. 5), in which two Na 8d site (shown as the red arrow in Fig. 5e) reside in the vicinity of the two of four Mo 8d sites with Na ordering on the shoulder of ellipse. When the Na/Fe_2_(MoO_4_)_3_ cell is discharged to 3/4 of the fully discharged state, the ABF image is different from one of any other ABF images for the pristine and partially sodiated Fe_2_(MoO_4_)_3_ at the 1/4 and 1/2 discharged states. In four spots representing Mo 8d sites on the shoulder of ellipse, three spots (yellow circles) are blacker than other one spot (white circles) in the Fig. 5c. They represent Na-occupied or Na-unoccupied in Na_1.5_Fe_2_(MoO_4_)_3_ structure based on first principle calculations (Supplementary Fig. 5) and the simulated ABF image (Fig. 5f). Na columns as red dash lines with the periodicity and homogeneity are also observed in all region of these images, indicating the feature of single domain. In the other word, ABF images at various discharge states not only characterize the solid solution mode of Na^+^ intercalation into Fe_2_(MoO_4_)_3_, but also provide the preferred occupancy patterns of Na^+^ in Fe_2_(MoO_4_)_3_ during the electrochemical intercalation process.

## Discussion

**Figure 6:**
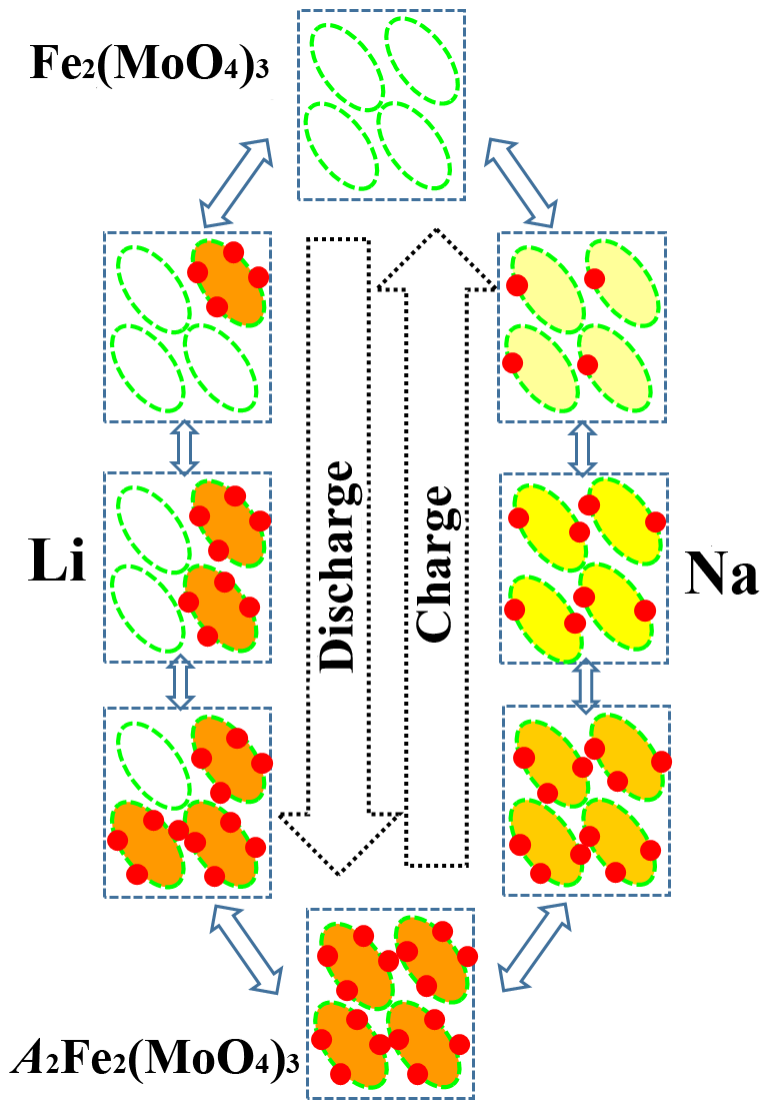
Comparison between sodiation and lithiation process in Fe_2_(MoO_4_)_3_. Schematic diagrams of “discrete occupation” and “pseudo-continuous occupation” during Li and Na ions intercalation into Fe_2_(MoO_4_)_3_. Solid red circles and dash green ellipses stand for Li^+^ (or Na^+^) and Fe_2_(MoO_4_)_3_ frameworks, respectively.

The high resolution atomic images provide a direct evidence on the different atomic occupancies of Li and Na in Fe_2_(MoO_4_)_3_ from the repeating Fe_2_(MoO_4_)_3_ unit of ellipse along the [001] projection in the partially lithiated and sodiated Fe_2_(MoO_4_)_3_. The existence of biphasic domains and the lithium occupancy near four of the Mo 8d sites taking a discrete manor (all empty or all occupied) during the discharge process of Li/Fe_2_(MoO_4_)_3_ cell are confirmed. The new formed Li_2_Fe_2_(MoO_4_)_3_ phase is revealed with four Li 8d sites occupying both sides of ellipse configuration in symmetric distribution apart from the original Fe_2_(MoO_4_)_3_ phase. In contrast, the single-phase domains with various compositions were observed at the different discharge states of Na/Fe_2_(MoO_4_)_3_ cell. Four Na 8d vacancies in the ellipse configuration are occupied one by one during the discharge process. According to the structural characteristics of Fe_2_(MoO_4_)_3_, its unit of ellipse along the [001] projection can hold four *A* atoms distributed on both sides of ellipse configuration. The electrochemical intercalated Li ions capture all four sites simultaneously to form Li_2_Fe_2_(MoO_4_)_3_ at the different discharge states. This feature can be depicted as a “discrete-occupation” path (shown in Fig. 6) that defines that the Li occupation for the four available 8a sites is either all empty or all occupied, in forming the Li_2_Fe_2_(MoO_4_)_3_. The amount of Li_2_Fe_2_(MoO_4_)_3_ phase increases with increasing Li content at the expense of Fe_2_(MoO_4_)_3_ phase. In contrast, Na ion intercalation in Fe_2_(MoO_4_)_3_ can be described as a “pseudo-continuous-occupation” path, where Na ions progressively occupy four of the 8d sites in the ellipse unit of Fe_2_(MoO_4_)_3_ one after another. The holistic occupation of Na ions in Fe_2_(MoO_4_)_3_ results in the formation of a series of pseudo continued Na*_x_*Fe_2_(MoO_4_)_3_ solid solutions in which *x* value increases from 0.0 to 2.0. Apparently, the different occupation behaviors of Li and Na ions in Fe_2_(MoO_4_)_3_ lead to two different structural change modes. Based on the *ex situ* XRD and electrochemical characterization results, the deintercalation of *A* ions from *A*_2_Fe_2_(MoO_4_)_3_ is reversible during the charge process.

The single-phase and two-phase modes of Fe_2_(MoO_4_)_3_ with the intercalation/deintercalation of Li and Na ions are revealed on atomic-scale. The orthorhombic Fe_2_(MoO_4_)_3_ electrode is a quite interesting material for studying the key factors governing the solid-solution and two-phase reactions during the ion intercalation/deintercalation process. Based on the calculated lattice information in Supplementary Table 2, the unit-cell volume changes of Fe_2_(MoO_4_)_3_ after the full lithiation and sodiation are ca. 2.96% and 4.72%, respectively. These values are significantly smaller than that of the LiFePO_4_ (6.8%)^8^. In addition, the calculated diffusion constants of Li^+^ and Na^+^ are 3.45 × 10^−8^ and 4.94 × 10^−11^ cm^2^s^−1^, respectively (Supplementary Fig. 6 and Supplementary Table 3). The smaller volume change and faster ion diffusion in the Li/Fe_2_(MoO_4_)_3_ cell contribute to its better cycle performance as compared with that in the Na/Fe_2_(MoO_4_)_3_ cell. Thus, Fe_2_(MoO_4_)_3_ electrode is also a good example to bridge the understanding of the relationship between the electrochemical properties and the intercalation/deintercalation process.

In summary, the two-phase and single-phase mechanisms were revealed during the intercalation/deintercalation of Li and Na ions into Fe_2_(MoO_4_)_3_. The “discrete occupation” and “pseudo-continuous” were proposed to describe the distinctly different occupation paths of Li and Na ions into Fe_2_(MoO_4_)_3_. The first-principle thermodynamic calculations and direct atomic-scale observation by STEM provide further insight on the intercalation/deintercalation process. Most importantly, the discrete occupation path for Li and pseudo-continuous occupation path for Na, and their relationship with two-phase reaction for Li and single-phase reaction for Na, respectively during the intercalation/deintercalation process in Fe_2_(MoO_4_)_3_ may very well be extended to the knowledge of other intercalation compounds. Noticeably, the present results were made at a relatively low rate of 1/20 C, which is close to the equilibrium state. The above mentioned intercalation/deintercalation reactions of of Li and Na ions into Fe_2_(MoO_4_)_3_ should be further investigated at the high rates which is far away from the equilibrium state in the future work. All in all, our experimental and theoretical studies could provide very valuable information for the research and development of intercalation compounds as electrode materials for secondary batteries.

## Methods

### Sample Preparation and Characterization

Orthorhombic Fe_2_(MoO_4_)_3_ microspheres were synthesized by a simple hydrothermal method^24^ using Fe(NO_3_)_3_·9H_2_O and Na_2_MoO_4_·2H_2_O as precursor. Scanning electron microscopy (SEM, Cambridge S-360) was employed to study the morphology and particle size of Fe_2_(MoO_4_)_3_ (Supplementary Fig. 7). Powder X-ray diffraction (XRD) patterns (Supplementary Fig. 8) were collected at beamline X14A of the National Synchrotron Light Source (NSLS) at Brookhaven National Laboratory using a linear position sensitive silicon detector. The wavelength used was 0.7747 Å. X-ray absorption spectroscopy (XAS) was performed at beamline X19A of the NSLS. Fe K-edge XAS was collected in transmission mode (Supplementary Fig. 9). The XAS data was processed using Athena^25^.

### Electrochemical measurements

A slurry of 80 wt % Fe_2_(MoO_4_)_3_, 10 wt % carbon black, and 10 wt % polyvinylidenefluoride (PVDF, Sigma-Aldrich) dispersed in N-methyl-2-pyrrolidone (NMP, Sigma-Aldrich) was prepared and cast on aluminum foil. The electrodes were dried at 120°C, and were punched to small circular pieces of diameter of 14 mm. Electrochemical cells were assembled into coin cells in an Ar-filled glovebox (MBraun, Germany). Sodium pieces and lithium pieces were used as a counter electrode for sodium and lithium batteries, respectively. The electrolytes consisted of 1 M NaPF_6_ (Alfa-Aesar) and LiPF_6_ (Alfa-Aesar) in a nonaqueous solution of ethylene carbonate (EC, Alfa-Aesar) and dimethyl carbonate (DMC, Alfa-Aesar) with a volume ratio of 1:1. Galvanostatic discharge-charge measurements were carried out at room temperature with a Land CT 2001A battery test system. The current densities and capacities of electrodes were calculated based on the weight of active materials. On the basis of two electrons transfer in the Fe_2_(MoO_4_)_3_, 1C was calculated to correspond to about 91.00 mA g^−1^.

### DFT simulations

All the first-principle total energy calculations were performed using a plane-wave basis set and the projector-augmented wave (PAW) method^26^ as implemented in the Vienna *ab initio* simulation package (VASP)^27^. Generalized gradient approximation (GGA) in the parametrization of Perdew, Burke, and Ernzerhof (PBE)^28^ pseudopotential was used to describe the exchange−correlation potential and a Hubbard-type correction U was taken into account due to the strongly correlated nature of the Fe 3d electrons^29^. Referring to the DFT calculations on LiFePO_4_ and FePO_4_ materials, the effective *U* value was set to 4.3 eV^30^. A kinetic energy cutoff of 550 eV was used in all calculations. Geometry optimizations were performed by using a conjugate gradient minimization until all the forces acting on ions were less than 0.02 eV/Å per atom. (1 × 2 × 2) and (1 × 1 × 2) Monkhorst-Pack grids were used for the bulk and interface supercells. Activation energies for Na^+^ and Li^+^ ion diffusion in Fe_2_(MoO_4_)_3_ were calculated using the nudged-elastic-band (NEB) method^31^ using seven images and two endpoint structures, which is a reliable method to search the minimum-energy path (MEP) when the initial and final states are known. An interpolated chain of configurations (images) between the initial and final positions are connected by springs and relaxed simultaneously to the minimum-energy path. The structure diagrams were drawn by VESTA^32^.

### STEM imaging

STEM samples were made by sonication of the discharged cathode films in anhydrous dimethyl carbonate inside an argon-filled glove box, and sealed in airtight bottles before immediately transfer into the STEM column. A JEM-ARM200F STEM operated at 200 KV and equipped with double aberration-correctors for both probe-forming and imaging lenses was used to perform high-angle annular-dark-field (HAADF) and ABF imaging. The attainable spatial resolution of the microscope is 78 pm at the incident semi-angle of 25 mrad. To observe Li directly using ABF collection geometry, the acceptance semi-angle in this study was fixed between 12 and 25 mrad. The STEM ABF and HAADF images were taken simultaneously at the optimal defocus value of the HAADF imaging condition, which was more defocused than the optimal ABF imaging condition on this instrument. Thus, the contrast in the ABF image is reversed with the bright area corresponding to the atomic positions^33^.

## Author Contributions

Z.-W.F. and S.-Q.S. planned the study and supervised all aspects of the research. J.-L.Y. and Z.-W.F. wrote the manuscript. J.-L.Y. and X.-Q.H. tested the electrochemical performance. J.-L.Y., J.L. and S.-Q.S. performed the DFT calculations. Y.-N.Z. and X.-Q.Y. performed the XRD and XAS measurements and analyzed the data. L.G. performed STEM observation and H.L., L.G., J.-L.Y., Z.S. and Z.-W.F. analyzed the STEM data. Z.-Z.Y. performed the STEM simulation. All the authors discussed the results and commented on the manuscript.

## Supplementary Material

Supplementary Information Supplementary information
