# Supplementary material for: Discrete Li-occupation versus pseudo-continuous Na-occupation and their relationship with structural change behaviors in Fe2(MoO4)3
Source: Sci Rep. 2015 Mar 6;5:8810. doi: 10.1038/srep08810 (PMC4351542; doi:10.1038/srep08810)
Supplement: Supplementary Information [file srep08810-s1.pdf]

Supplementary Information

Discrete Li-occupation versus pseudo-continuous Na-occupation and their relationship with structural change behaviors in $Fe_2(MoO_4)_3$

Ji-Li Yue[1], Yong-Ning Zhou[2], Si-Qi Shi[3*], Zulipiya Shadike[1], Xuan-Qi Huang[1], Jun Luo[3], Zhen-Zhong Yang[4], Hong Li [4], Lin Gu[4*], Xiao-Qing Yang[2], Zheng-Wen Fu[1*]

1. Shanghai Key Laboratory of Molecular Catalysts and Innovative Materials, Department of Chemistry & Laser Chemistry Institute, Fudan University, Shanghai 200433, P. R. China
2. Chemistry Department, Brookhaven National Laboratory, Upton, New York 11973, USA
3. School of Materials Science and Engineering, Shanghai University, Shanghai 200444, P. R. China
4. Beijing National Laboratory for Condensed Matter Physics, Institute of Physics, Chinese Academy of Sciences, PO Box 603, Beijing 100190, P. R. China

Ji-Li Yue and Yong-Ning Zhou contribute equally to this work

**E-mail:*sqshi@shu.edu.cn;*l.gu@aphy.iphy.ac.cn;zwfu@fudan.edu.cn*

Supplementary Figures

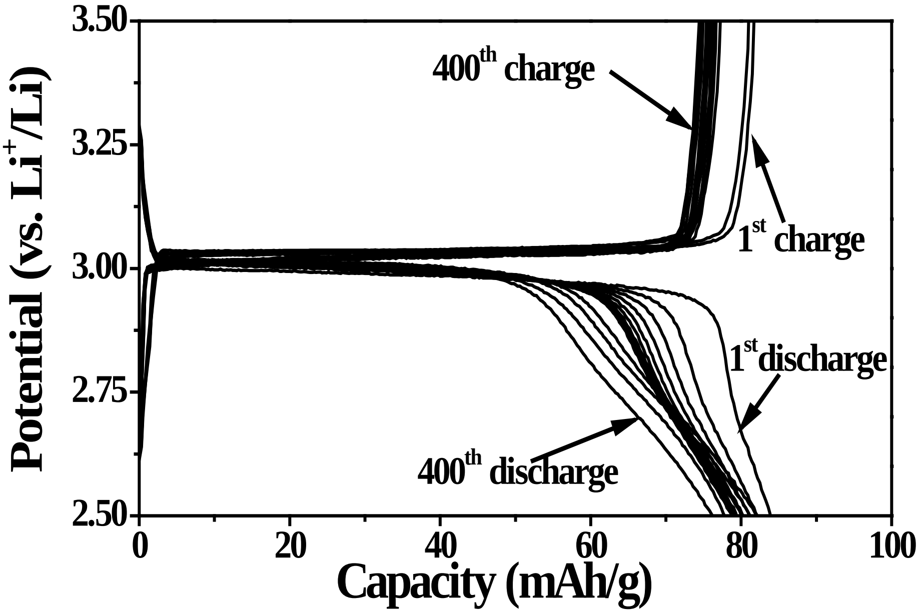

Supplementary Figure 1 | Electrochemical performance. The selected galvanostatic discharge/charge curves of a Li/$Fe_2(MoO_4)_3$ cell at the 1st-10th,100th, 200th, 300th and 400th cycles at a current density of C/5.

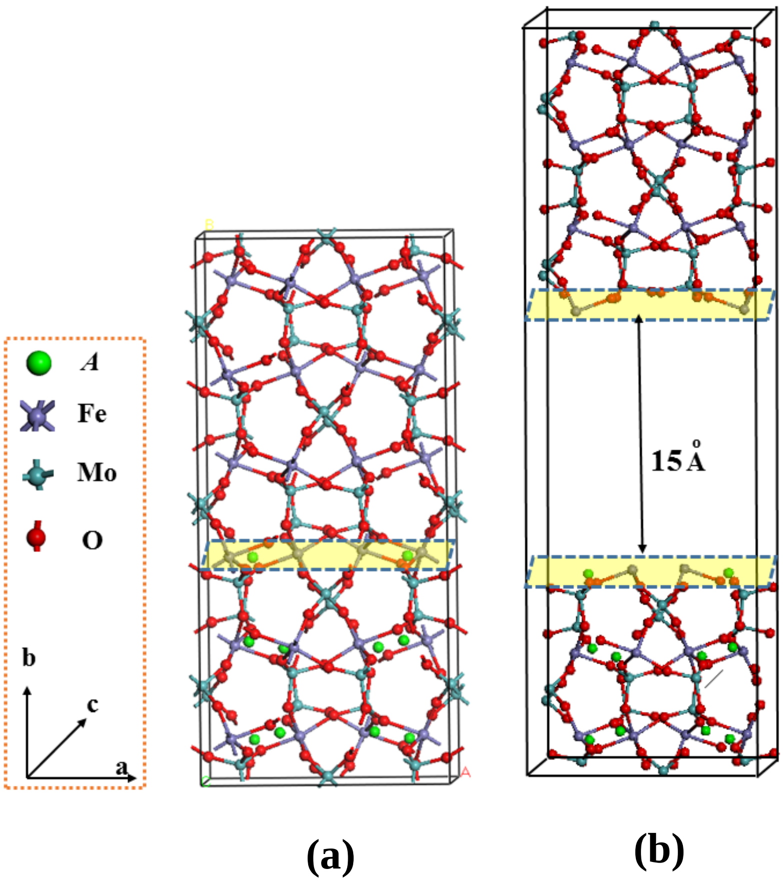

Supplementary Figure 2 | A model for an $A_2Fe_2(MoO_4)_3/Fe_2(MoO_4)_3$ interface. Relaxed crystal structure of (a) bulk $Fe_2(MoO_4)_3$ supercell and (b) one possible (010) plane supercell including one vacuum layerand two bulk crystal slabs with *A*-contained part (bottom slab) and *A*-free part (top slab). Actually, there are two identical interfaces, but only one is shown.

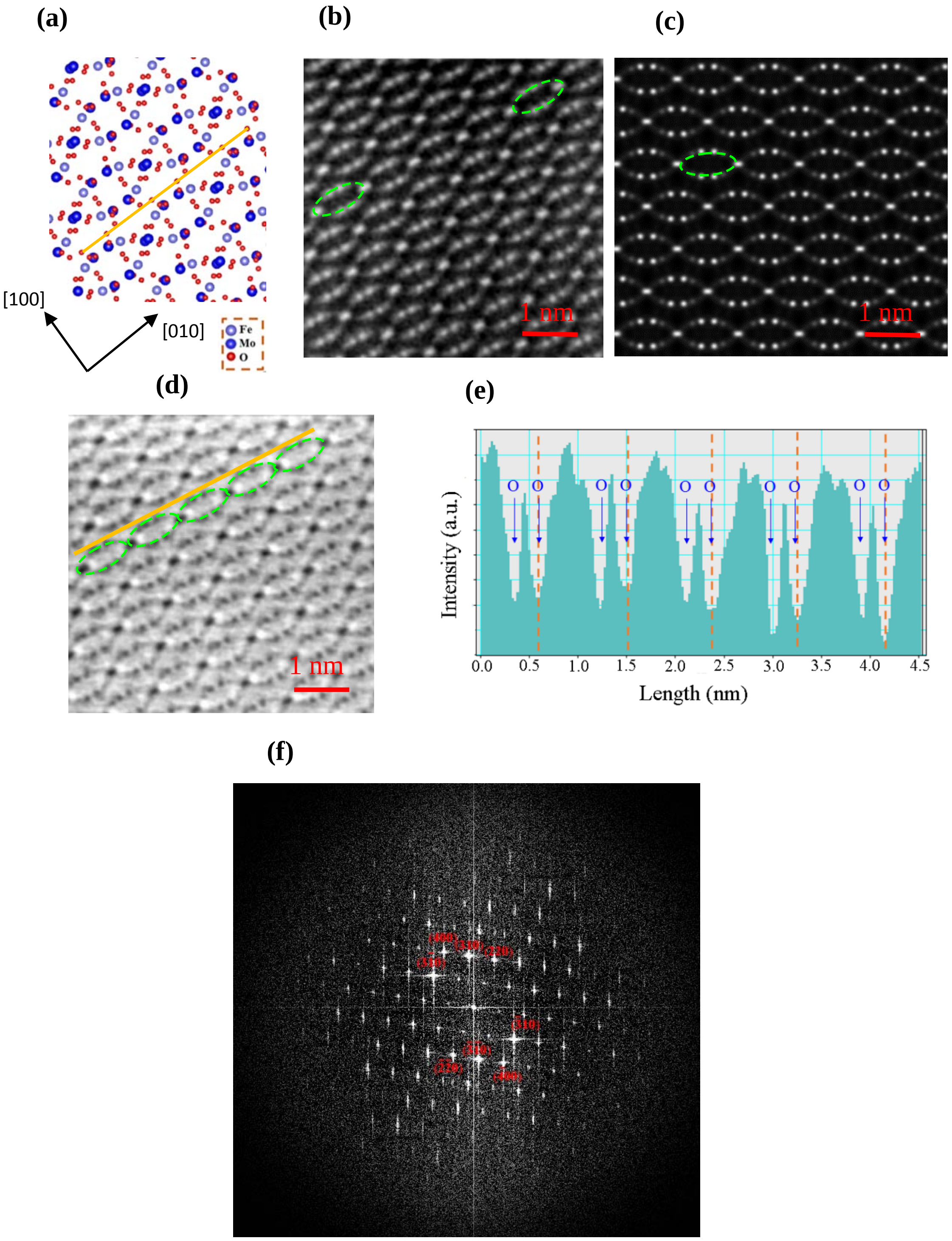

Supplementary Figure 3 | STEM imaging of pristine $Fe_2(MoO_4)_3$. (a) Schematic drawing of $Fe_2(MoO_4)_3$ lattice, (b) the experimental and (c) simulated HAADF STEM image for $Fe_2(MoO_4)_3$ along the [001] zone axis, (d) the ABF STEM image and (e) the corresponding line profile of ABF to Figure (d) acquired at the yellow line, and the O sites are marked by blue arrows, (f) the corresponding fast Fourier transform (FFT) of the ABF STEM image.

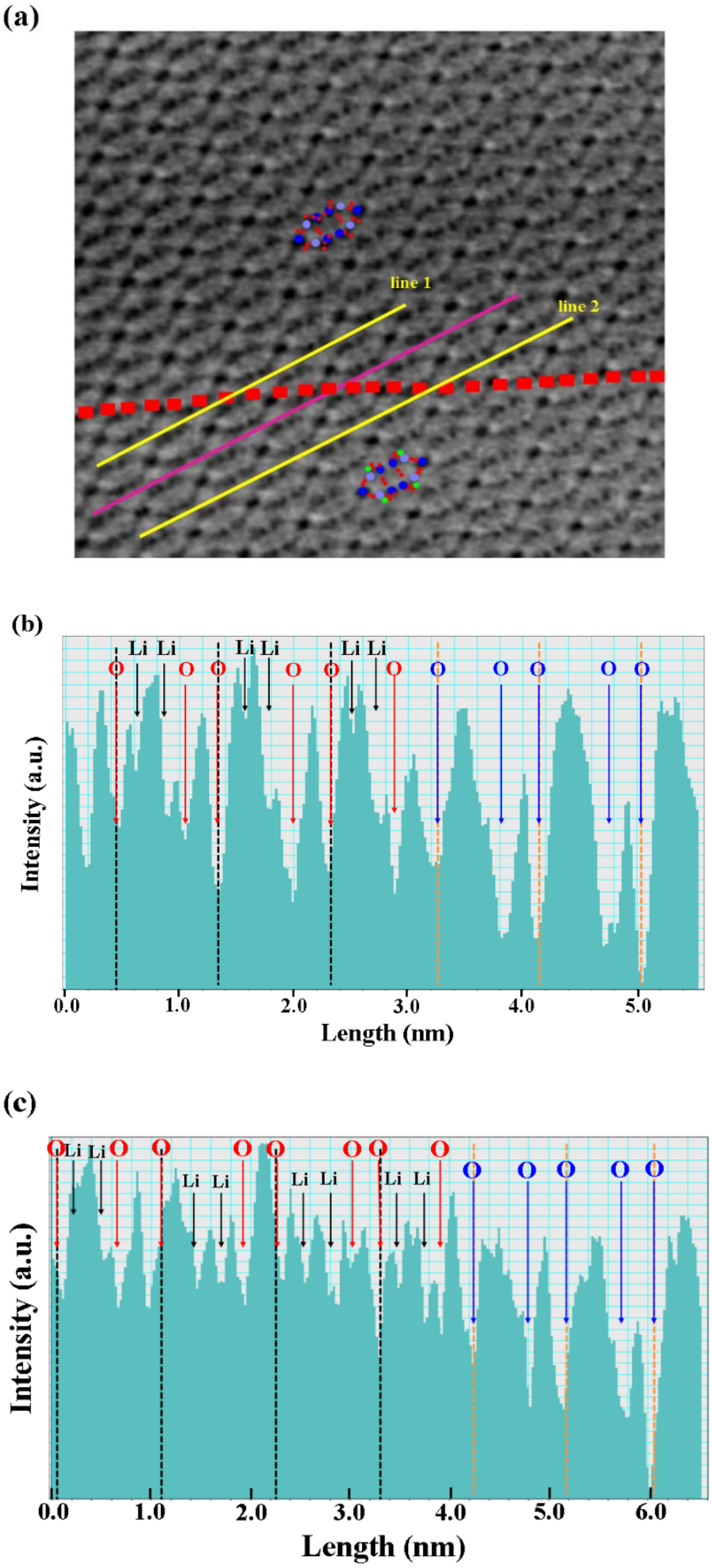

Supplementary Figure 4 | Two phase Interface. (a) ABF-STEM image of partially lithiated $Fe_2(MoO_4)_3$ at the 1/2 discharge state and the corresponding ABF line profile of yellow line 1 (b) and 2 (c) in (a).

(a)

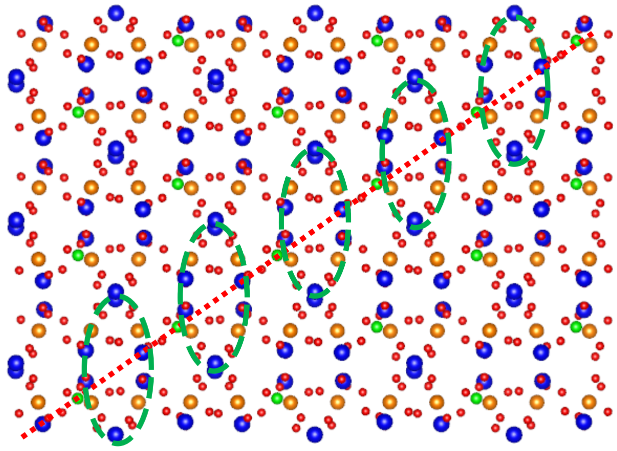

(b)

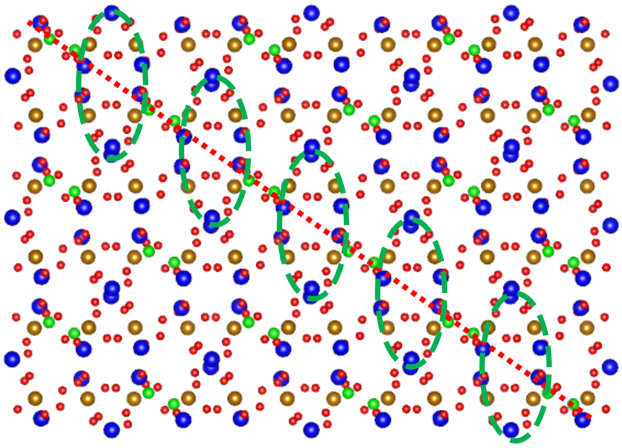

(c)

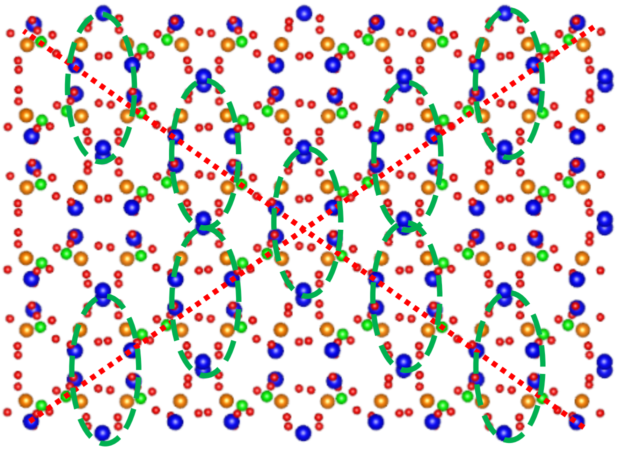

Supplementary Figure 5 | The energetically most favorable configurations of partially sodiated $Fe_2(MoO_4)_3$ from the first principles calculations. (a) $Na_{0.5}Fe_2(MoO_4)_3$; (b) $Na_{1.0}Fe_2(MoO_4)_3$; (c) $Na_{1.5}Fe_2(MoO_4)_3$ viewed along the [001] direction. Green, brown, blue and red balls stand for Na, Fe, Mo and O ions, respectively.

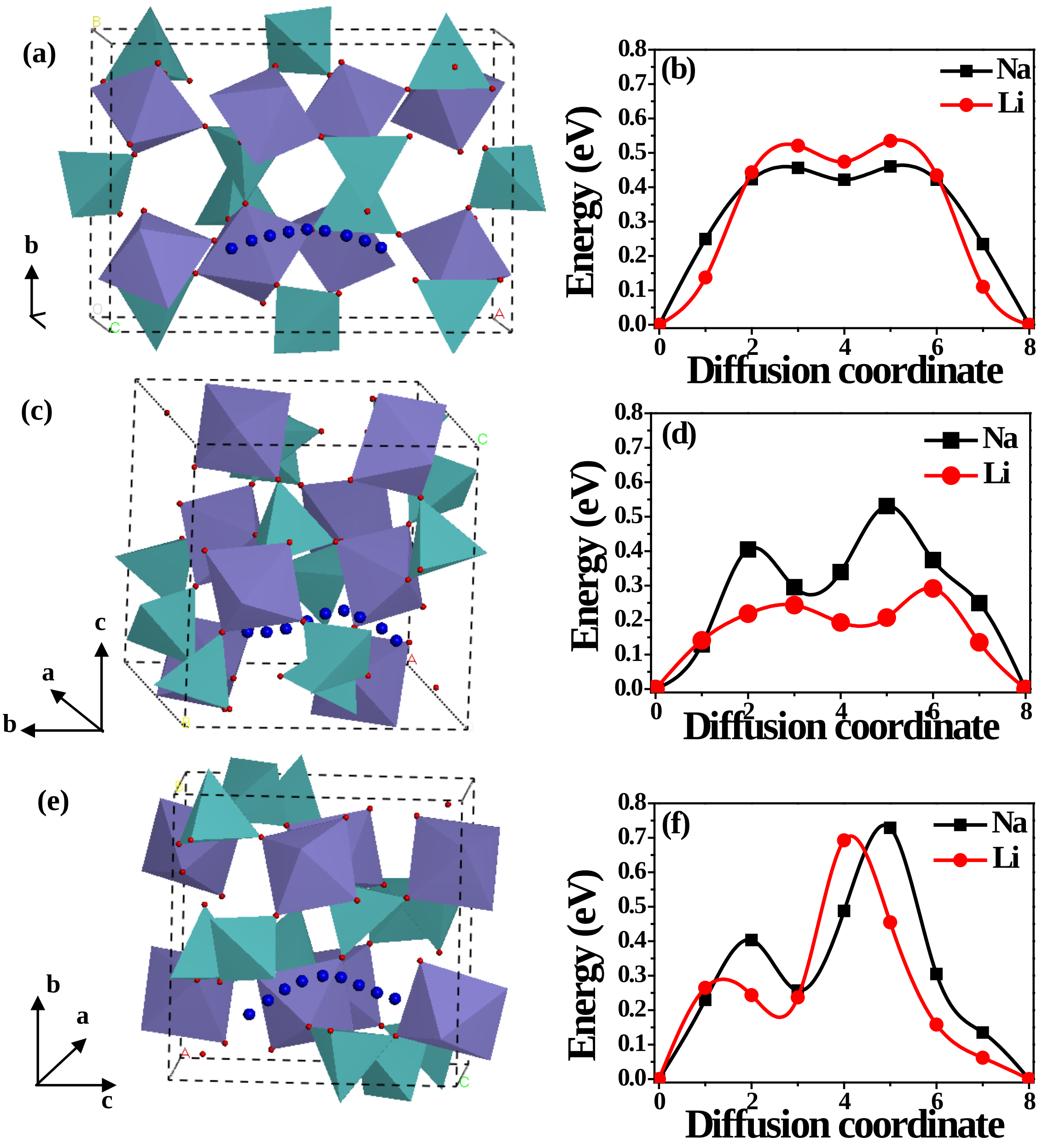

Supplementary Figure 6 | Three possible diffusion paths and corresponding energy profiles. Along the (a, b) [100], (c, d) [010] and (e, f) [001] directions of A^+ in $Fe_2(MoO_4)_3$. Purple octahedras and green tetrahedras stand for FeO_6 and MoO_4 respectively, and blue balls stand for the calculated A^+ diffusion trajectory.

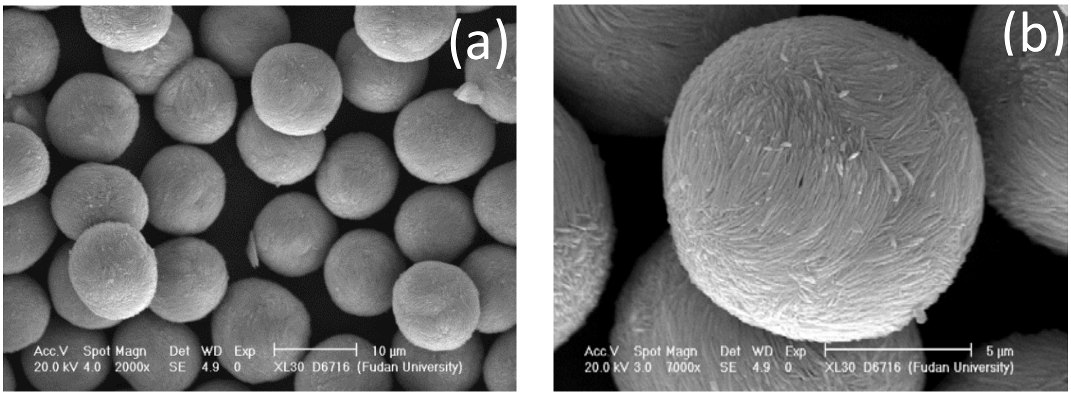

Supplementary Figure 7 | SEM images. SEM images of the hydrothermal synthetic $Fe_2(MoO_4)_3$ sample with magnification of (a) 2000× and (b) 7000×.

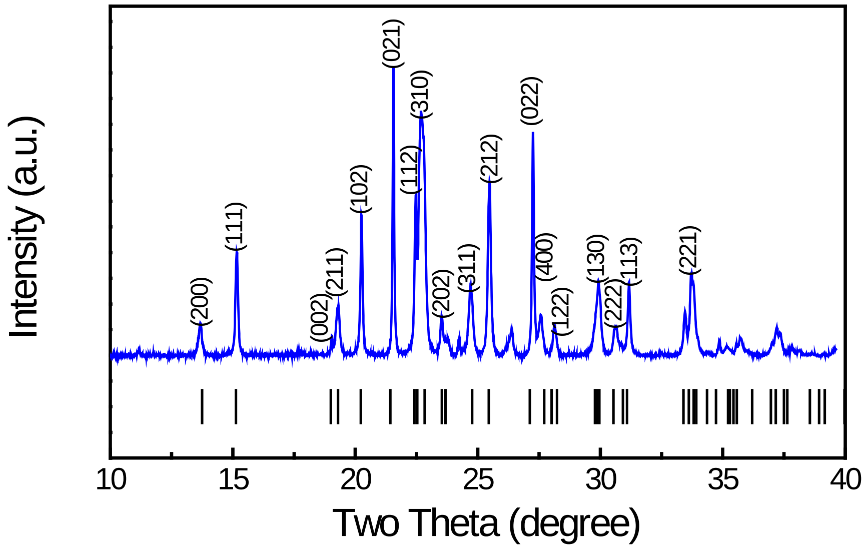

Supplementary Figure 8 | XRD pattern. XRD pattern of the pristine $Fe_2(MoO_4)_3$ powder synthesized by the hydrothermal method. All peaks can be well indexed to an orthorhombic structure with a space group of Pbcn (JCPDS No. 852287)**.**

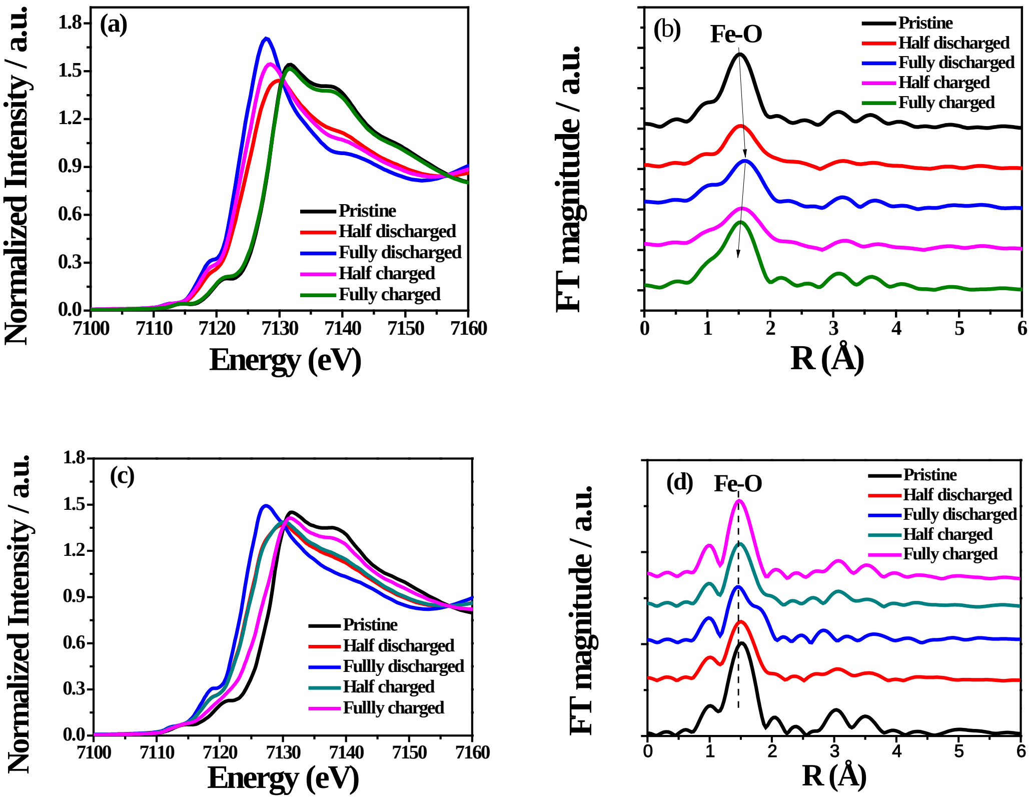

Supplementary Figure 9 | Fe K-edge XANES spectra. (a) Normalized Fe K-edge XANES spectra and (b) k^2-weighted Fourier transform magnitudes of Fe K-edge EXAFS spectra of the pristine (x=0), half discharged (x=1),full discharged (x=2), half charged (x=1) and full charged (x=0) $Li_xFe_2(MoO_4)_3$. (c) Normalized Fe K-edge XANES spectra and (d) k^2-weighted Fourier transform magnitudes of Fe K-edge EXAFS spectra of the pristine (x=0), half discharged (x=1),full discharged (x=2), half charged (x=1) and full charged (x=0) $Na_xFe_2(MoO_4)_3$.

Supplementary Table 1 | Various phase transition behaviors of reported intercalation materials for lithium and sodium batteries.

Host material	Guest ion	Phase transition behavior	References
$LiFePO_4$	Li^+	Two-phase	1
$Li_4Ti_5O_{12}$	Li^+	Two-phase	2
$Li_4Ti_5O_{12}$	Na^+	Three-phase	3
$Li_xCoO_2(0.5<x\leq0.75)$	Li^+	Single-phase	4
$Na_xCoO_2(0.5\leq x\leq1)$	Na^+	Many single/two phase domains	5
$Li_2CoP_2O_7$	Li^+	Single-phase	6
$Li_3V_2(PO_4)_3$	Li^+	Two-phase	7
$Li_{1.32}Mn[Fe(CN)_6]_{0.83}$	Li^+	Single-phase	8
Li_2CoSiO_4	Li^+	Two-phase	9
$NaCrO_2$	Na^+	Many single-/two-phase domains	10
$Na_3V_2(PO_4)_3$	Na^+	Two-phase	11
$NaTi_2(PO_4)_3$	Na^+	Two-phase	12
$Na_2Fe_2(SO_4)_3$	Na^+	Single-phase	13

Supplementary Table 2 | Lattice parameters and unit cell volumes of the pristine and fully discharged phases for $Fe_2(MoO_4)_3$ calculated from the XRD patterns.

	a (Å)	*b*(Å)	*c*(Å)	*V* (Å^3)	*ΔV*
$Fe_2(MoO_4)_3$	12.856	9.238	9.325	1107.5	-
$Li_2Fe_2(MoO_4)_3$	12.872	9.344	9.481	1140.3	2.96%
$Na_2Fe_2(MoO_4)_3$	12.983	9.375	9.528	1159.7	4.72%

Supplementary Table 3 | Calculated activation barriers (E_{act}, Supplementary Fig. 6b, d and f) and diffusion constants (D) for A^+ hopping along the three directions.

A^+	direction	E_{act}(eV)	D (cm^2s^{-1})
Li^+	[100]	0.53	3.08×10^{-12}
	[010]	0.29	3.45×10^{-8}
	[001]	0.69	6.51×10^{-15}
Na^+	[100]	0.46	4.94×10^{-11}
	[010]	0.53	3.33×10^{-12}
	[001]	0.73	1.48×10^{-15}

Supplementary Notes

Supplementary Figure 2. Periodic boundary conditions were used, so each phase is sandwiched by the other phase and the system has two identical interfaces in the supercell. Due to the lattice mismatch of 1.6% (2.1%) between the $Li_2Fe_2(MoO_4)_3$ ($Na_2Fe_2(MoO_4)_3$) and $Fe_2(MoO_4)_3$ phases along the [010] direction, the latter was stretched to match the former to produce a three-dimensional periodic bulk-like interface system under coherent interface approximation. Considering the stoichiometric cleaving, and the Mo-O bonds should not be cleaved, the Li-O bonds and Fe-O bonds are cleaved. The termination plane (*x*, 0.25, *z*) and (*x*, 0.75, *z*) for the (010) surface is energetically favorable. A vacuum layer of 15 Å is used to be enough to remove any spurious interaction between the periodically repeated slabs along the [010] direction. Calculations of $E_{A,\mathrm{contained}}$ and $E_{A,\mathrm{free}}$ can be carried out by building this vacuum slab between the *A*-contained part and *A*-free part, creating a supercell. In the interface system, atoms in the interior of each phase are frozen to reproduce bulk behavior whereas atoms in the interface region are relaxed.

Supplementary Figure 3. The contrast of the annular-bright-field (ABF) image exhibits a $Z^{1/3}$ dependency in contrast to the $Z^{1.7}$ dependency for high-angle annular-dark-field (HAADF) imaging, where Z represents the atomic number. Based on the crystal structure obtained from first principles calculations[2,3]. (Supplementary Fig. 3a), the light element of O is almost indiscernible and heavy elements of Mo and Fe can be clearly visible in the HAADF and ABF images along the [001] projection as shown in supplementary Fig. 3 b and d, respectively. The repeated unit can be clearly visualized (shown in the green ellipses in the inset). In Supplementary Fig. 3b, the strong white spots in both tips of ellipses represent Mo 4c sites along the [001] projection. Other four brightly white spots and four weakly white spots symmetrically distributed in ellipses represent Mo 8d sites and Fe 8d sites along the [001] projection, respectively. The patterns are in good agreement with the atomic occupancies of simulated HAADF in Supplementary Fig. 3c. In the Supplementary Fig. 3d, four spots representing Mo 8d sites at every repeated unit structure labelled by green ellipse exhibit the same black. The corresponding line profile of ABF to Supplementary Fig. 3d acquired at the yellow line shows the periodic characteristics of oxygen occupation as shown in Supplementary Figure 3e. The FFT results (see Supplementary Figure 3f) confirm that the material is pure orthorhombic $Fe_2(MoO_4)_3$ during STEM analysis process.

Supplementary Figure 7. The as-prepared samples are composed of mainly uniformly spherical aggregates with a size of about ten micrometers. Detailed surface observation implied that each microsphere is constructed by elongated rods, which are about eight hundred nanometers in diameter and five micrometers in length.

Supplementary Figure 9. When the $Na/Fe_2(MoO_4)_3$ cell is discharged, the shift of Fe K-edge absorption energy toward low energy is observed as shown in supplementary

Fig. 9a. The Fe K-edge at 7125.7 eV for the pristine $Fe_2(MoO_4)_3$ indicates the valence state of Fe is +3[14]. During the discharge process of the Li/$Fe_2(MoO_4)_3$ cell. Fe K-edge gradually shift toward lower energy and locates at 7120.8 eV after discharging to 2.5 V, which can be attributed to that of $Li_2Fe_2(MoO_4)_3$ with Fe^{2+}[14], indicating the reduction of iron from Fe^{3+} to Fe^{2+}. During the charging process, Fe K-edge absorption energy shifts back toward higher energy. The XANES spectra can fully recover its pristine state after recharging to 3.5 V, indicating the excellent reversibility.

Supplementary Fig. 9b shows the Fourier transform magnitudes of Fe K-edge EXAFS spectrum at different states. The peaks at around 1.5 Å is related to Fe-O bond. The spectrum demonstrates a reversible local structure change around Fe atoms during discharge and charge process. For the Na/$Fe_2(MoO_4)_3$ cell, the shift of Fe K-edge in Supplementary Fig. 9c and the change of FT-EXAFS spectra in supplementary Fig. 9d are similar as those of the Li/$Fe_2(MoO_4)_3$ cell, but the spectra after fully charge are not exactly same as those of the pristine state, indicating a little poor reversibility compare to Li/$Fe_2(MoO_4)_3$ cell.

Supplementary Table 3. D is calculated from the equation[15] $D = a^2 v \exp(-E_{act}/k_B T)$, where a is the hopping distances between the neighboring two Li^+(Na^+) sites in $Li_2Fe_2(MoO_4)_3$ ($Na_2Fe_2(MoO_4)_3$), which is 4.969 (5.135)Å, 5.071 (5.161) Å or 5.042 (5.202) Å along the [100], [010] or [001] direction, v is the lattice vibration frequency and a typical value of 10^{12} Hz is used here; k_B is the Boltzmann constant; T is the room temperature (300K); E_{act} is the diffusion barrier which is obtained from the NEB results.

The minimum energy pathways for A^+ along the [100], [010] and [001] directions are depicted in supplementary Fig. 6(a), (c) and (e), indicating a curved trajectory between A^+ sites (rather than the direct linear jump). These are similar to the situation of $LiFePO_4$ that the Li^+ diffusion trajectory is also a curved path which is elucidate by the first principle calculations[16] and neutron diffraction study[17].The calculated Li^+ migration barriers along the [100], [010] and [001] directions are 0.53, 0.29 and 0.69 eV, respectively. Accordingly, Li^+ diffusion constant of 3.45×10^{-8} cm^2 s^{-1} (see Supplementary Table 3) can be obtained from a lowest-energy pathway (0.29 eV) for Li^+ migration along the [010] direction, which is close to $LiFePO_4$ (10^{-7} cm^2 s^{-1}) [15]. Na^+ migration barriers along the [100], [010] and [001] directions are 0.46, 0.53 and 0.73 eV, respectively. Therefore, Na^+ diffusion constants along [100] and [010] directions are 4.94×10^{-11} and 3.33×10^{-12} cm^2 s^{-1} (see Supplementary Table 3), respectively.

Supplementary References

1. Padhi, A. K., Nanjundaswaswamy, K. S. & Goodenough, J. B. Phospho-olivines as positive-electrode materials for rechargeable lithium batteries. *J. Electrochem. Soc* **144**, 1188-1194 (1997).
2. Lu, X. et al. Lithium storage in $Li_4Ti_5O_{12}$ spinel: the full static picture from electron microscopy. *Adv. Mater.* **24**, 3233-3238 (2012).
3. Sun, Y. et al. Direct atomic-scale confirmation of three-phase storage mechanism in $Li_4Ti_5O_{12}$ for room-temperature sodium-ion batteries. *Nat. Commun.* **4**, 1870 (2013).
4. Mizushima, K., Jones, P.C., Wiseman, P. J. & Goodenough, J. B. Li_xCoO_2 ($0<x\leq1$): A new cathode material for batteries of high energy density. *Mater. Res. Bull.* **15**, 783 – 789 (1980).
5. Berthelot, R., Carlier, D. & Delmas, C., Electrochemical investigation of the P2–Na_xCoO_2 phase diagram. *Nat. Mater.* **10**, 74-80 (2011).
6. Shakoor, R. A. et al. Site-specific transition metal occupation in multicomponent pyrophosphate for improved electrochemical and thermal properties in lithium battery cathodes: a combined experimental and theoretical study. *J. Am. Chem. Soc.* **134**, 11740-11748 (2012).
7. Yin, S. C. et al. Electrochemical property: structure relationships in monoclinic $Li_{3-y}V_2(PO_4)_3$. *J. Am. Chem. Soc.* **125**, 10402–10411 (2003).
8. Moritomo, Y., Takachi, M., Kurihara, Y. & Matsuda, T. Synchrotron-Radiation X-Ray investigation of Li^+/Na^+ intercalation into prussian blue analogues. *Adv. Mater. Sci. Eng.* **2013**, 967285 (2013).
9. He, G., Popov, G. & Nazar, L. F. Hydrothermal synthesis and electrochemical properties of Li_2CoSiO_4/C nanospheres. *Chem. Mater.* **25**, 1024-1031 (2013).
10. Zhou, Y. N. et al. Phase transition behavior of $NaCrO_2$ during sodium extraction studied by synchrotron-based X-ray diffraction and absorption spectroscopy. *J. Mater. Chem. A***1**, 11130-11134 (2013).
11. Jian, Z. L. et al. Superior electrochemical performance and storage mechanism of $Na_3V_2(PO_4)_3$ cathode for room-temperature sodium-ion batteries. *Adv. Energy Mater.* **3**, 156-160 (2013).
12. Senguttuvan, P. et al. Low-potential sodium insertion in a NASICON-Type Structure through the Ti(III)/Ti(II) redox couple. *J. Am. Chem. Soc.* **135**, 3897-3903 (2013).
13. Barpanda, P. et al. A 3.8-V earth-abundant sodium battery electrode. *Nat. Commun.* **5**, 1-8 (2014).
14. Shirakawa, J., Nakayama, M., Wakihara, M. & Uchimoto, Y. Changes in electronic structure upon lithium insertion into $Fe_2(SO_4)_3$ and $Fe_2(MoO_4)_3$ investigated by X-ray absorption spectroscopy. *J Phys. Chem. B* **111**, 1424-1430 (2007).
15.Morgan, D., Van der Ven, A. & Ceder, G. Li conductivity in Li_xMPO_4 (M=*Mn, Fe, Co, Ni*) olivine materials. *Electrochem. Solid State Lett.* **7**, A30-A32 (2004).
16. Islam, M. S., Driscoll, D. J., Fisher, C. A. J.& Slater, P. R. Atomic-scale investigation of defects, dopants, and lithium transport in the $LiFePO_4$ olivine-type battery material. *Chem. Mater.* **17**, 5085-2092 (2005).
17. Nishimura, S. I. et al. A Experimental visualization of lithium diffusion in Li_xFePO_4. *Nat. Mater.* **7**, 707-711 (2008).
